# Pre-therapy liver transcriptome landscape in Indian and French patients with severe alcoholic hepatitis and steroid responsiveness

**DOI:** 10.1038/s41598-017-07161-4

**Published:** 2017-07-28

**Authors:** Shvetank Sharma, Jaswinder S. Maras, Sukanta Das, Shabir Hussain, Ashwani K. Mishra, Saggere M. Shasthry, Chhagan B. Sharma, Emmanuel Weiss, Laure Elkrief, Pierre-Emmanuel Rautou, Hélène Gilgenkrantz, Sophie Lotersztajn, Valérie Paradis, Pierre de la Grange, Christophe Junot, Richard Moreau, Shiv K. Sarin

**Affiliations:** 1 Department of Molecular and Cellular Medicine, Institute of Liver and Biliary Sciences, New Delhi, India; 2 NexGenBio Life Sciences, New Delhi, India; 3 Department of Hepatology, Institute of Liver and Biliary Sciences, New Delhi, India; 4 Department of Pathology, Institute of Liver and Biliary Sciences, New Delhi, India; 5 INSERM, Université Paris Diderot, CNRS, Centre de Recherche sur l’Inflammation (CRI), Paris, France; 6 Département Hospitalo-Universitaire (DHU) UNITY, Service d’Hépatologie, Hôpital Beaujon, Assistance Publique-Hôpitaux de Paris, Clichy, France; 7 INSERM, Université Paris Descartes, CNRS, Institut Cochin, Paris, France; 8 Département de D’Anatomopathologie, Hôpital Beaujon, Assistance Publique-Hôpitaux de Paris, Clichy, France; 9 Genosplice, Institut du Cerveau et de la Moelle épinière ICM, Paris, France; 10 Laboratoire d’Etude du Métabolisme des Médicaments, DSV/iBiTec-S/SPI, CEA-Saclay, MetaboHUB-Paris, Paris, France; 11 Laboratoire d’Excellence Inflamex, COMUE Sorbonne Paris Cité, Paris, France

## Abstract

Patients with severe alcoholic hepatitis (SAH) not responding to glucocorticoid therapy have higher mortality, though they do not differ in their baseline clinical characteristics and prognostic scores from those who respond to therapy. We hypothesized that the baseline hepatic gene expression differs between responders (R) and non-responders (NR). Baseline liver transcriptome was compared between R and NR in Indian (16 each) and French (5 NR, 3 R) patients with SAH. There were differentially expressed genes (DEGs) between NR and R, in Indian (1106 over-expressed, 96 under-expressed genes) and French patients (65 over-expressed, 142 under-expressed genes). Indian NR had features of hepatocyte senescence and French NR exhibited under-expression of genes involved in cell division, indicating a central defect in the capacity of hepatocytes for self-renewal in both populations. Markers of hepatic progenitor cell proliferation were either very few (Indian patients) or absent (French patients). No DEGs were enriched in inflammatory pathways and there were no differences in nuclear receptor subfamily 3 group C member 1 (*NR3C1*) transcript expression and splicing between NR and R. Our results reveal that baseline hepatic transcriptome is reflective of subsequent glucocorticoid non-response and indicate impaired regenerative potential of the liver as an underlying phenomenon in NR.

## Introduction

Patients with severe alcoholic hepatitis (SAH) are usually treated with glucocorticoids, prednisolone, at a dose of 40 mg per day^1^. The Lille score is used to evaluate the response to glucocorticoid therapy at day 7. Patients who are responders (R) at day 7 continue to be treated with prednisone for 3 more weeks while in the non-responders (NR) do not continue the treatment^2, 3^. It is important to note that there is no established therapeutic alternative for NR. It has been suggested that “salvage” liver transplantation could be used in very selected NR, but this therapeutic option is controversial^4^. Mortality is significantly higher in NR than in R^1^; the reasons why the disease is more severe in NR than R are unclear. We hypothesized that at baseline marked differences in the underlying “sub-clinical” mechanisms of liver injury exist between NR and R. An appropriate approach to address this hypothesis is to perform a comprehensive analysis of the liver transcriptome in patients with SAH before any treatment. In addition, the potential advantage of this kind of study is to identify “pathophysiology-based” targets for novel therapeutic approaches. Since there has been no such study yet, we decided to use high throughput sequencing in order to describe the baseline transcriptome landscape in livers from patients who will respond to glucocorticoids and in those who will not. Since SAH is a global disease, in this pilot study, we investigated patients from India and also patients from France, so as to see if the observations have universal relevance.

## Results

### Patient characteristics

#### Indian patients

There were no major differences between NR and R at baseline in the clinical and biological characteristics (Table S1). Only leukocyte and platelet counts were significantly higher at baseline in NR as compared to R. As expected^5^, 1-month mortality rate was higher in NR than in R. Regarding liver pathology, the only differences between NR and R were higher scores of ballooning and Mallory-Denk bodies in NR.

#### French patients

There were no major differences between NR and R at baseline in the clinical and biological characteristics (Table S1). Only MELD score and MDF were significantly higher in NR than R. One-month mortality rate tended to be higher in NR than in R. Regarding liver pathology, the only differences between NR and R were higher Matavir score and lower ballooning score in NR (Table S1).

### Baseline liver transcriptome: Livers vs. PBMCs

#### Indian patients

From RNA-Seq analysis of the livers and PBMC, a total of 40,453 targets were identified (including mRNA, tRNA, miscRNA, snoRNA, ncRNA and pseudogenes), both containing 19,805 protein-coding genes. We obtained an average of 36.9 × 10^6^ (23 × 10^6^–74 × 10^6^) and 37 × 10^6^ (23.3 × 10^6^–74.7 × 10^6^) reads/sample in liver and PBMC respectively (Figure S1).

#### French patients

The expression microarray analysis of the livers identified 23,361 transcripts, whereas RNA-seq (with an average of 48.1 × 10^6^ reads/sample) identified 16,498 transcripts. There was a significant correlation between the gene expression profile obtained from microarray and RNA-seq analysis (Figure S2), and henceforth the microarray results were used for comparative analysis between the two populations. Expression microarray analysis of PBMC identified 25,939 transcripts.

#### Comparative analysis

**Figure 1:**
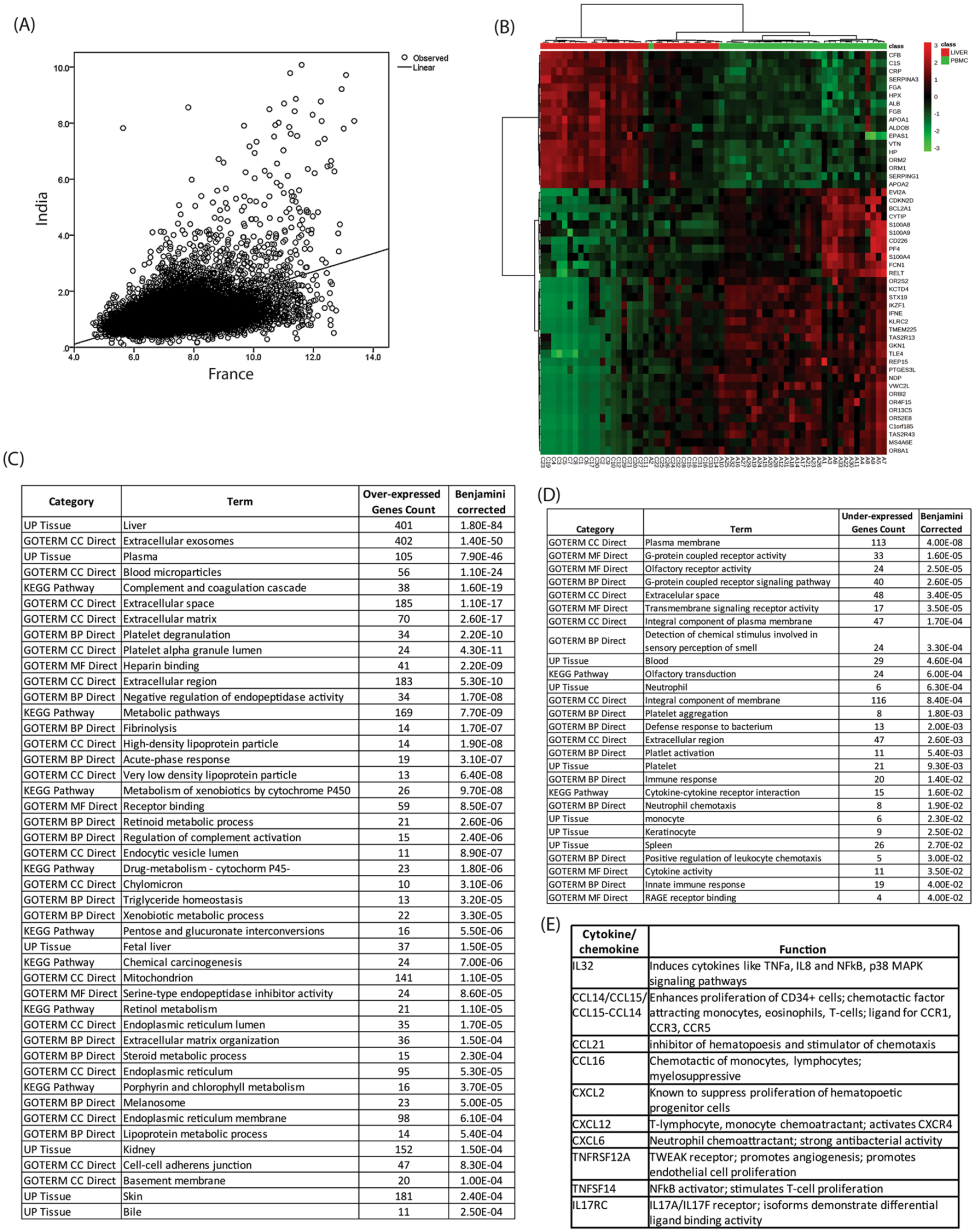
Comparative analysis of liver and PBMC identify common and distinct signatures in Indian and French SAH patients. (**A**) Correlation of baseline hepatic gene expression by microarray and RNA-Seq in French and Indian series, respectively (*r*_*s*_ = 0.374, p < 0.0001). (**B**) Heatmap based on top 50 differentially expressed genes between livers and PBMCs in Indian cohort. Enrichment analysis of DEGs that were over-expressed (**C**) and under-expressed (**D**) between the two tissues. (**E**) Cytokines/chemokines that were over expressed and were common between the two cohorts of patients.

There was a significant correlation between gene expression captured by microarray and RNA-seq in the two populations (*r*_*s*_ = 0.374, p < 0.001, Fig. 1A). The transcripts from each series of livers, Indian and French, were compared with the respective PBMC transcripts and DEGs were identified (Fig. 1B and Table S2, for DEGs in Indian patients; Table S3A,B for French patients). The number of DEGs differed between the Indian and French patients (1,663 vs. 8,872, respectively). However, functional analysis revealed that, in both Indian and French patients, genes that were over-expressed in the livers as compared to PBMCs were enriched the UP_TISSUE term “Liver” and in similar GO terms and KEGG pathways related to major hepatic functions (Coagulation and complement cascades, Xenobiotic metabolism process) (Fig. 1C and Table S3C). Moreover, genes that were underexpressed in livers were enriched in the UP_TISSUE term “Blood”, “Monocyte”, “Neutrophil” and in pathways that are characteristics of mononuclear cells present in the blood (Inflammatory response, innate immune response) (Fig. 1D and Table S3D). Together these findings show that the two techniques used in this study were able to capture “liver-specific” genes and describe the baseline liver transcriptome in patients with SAH. Interestingly, we found that some cytokines, chemokines and cytokine receptors were over-expressed in both Indian and French livers (Fig. 1E).

### Comparison of baseline liver transcriptome between NR and R

#### Indian livers

**Figure 2:**
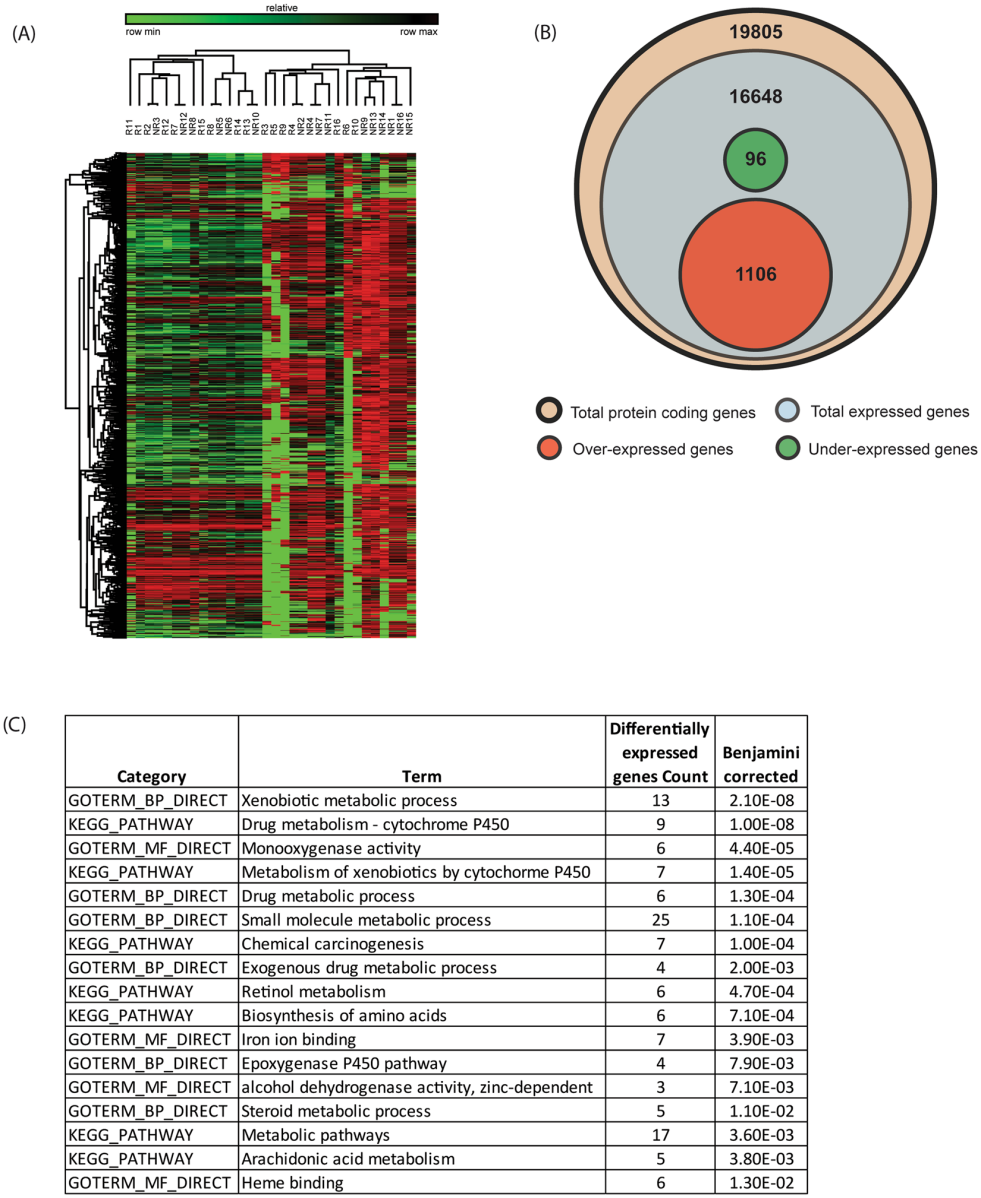
NR vs. R in livers from Indian patients. (**A**) Heat map of genes that were significantly and differentially expressed between NR and R. (**B**) Venn diagram showing the distribution of total genes, total protein coding genes, and differentially up- or down-regulated protein coding genes among NR. (**C**) Functional enrichment based on gene ontology analysis of under-expressewd genes in NR.

We found 1,202 DEGs between livers from NR and R; 1,106 were over-expressed and 96 under-expressed in NR relative to R (Fig. 2A,B). Finally, 16,102 genes were expressed but not differentially regulated between NR and R (Fig. 2B and Table S4B).

Using DAVID, we found that over-expressed genes did not exhibit enrichment for any particular functional category suggesting that these genes are related to many diverse functions. In contrast, under-expressed genes were significantly enriched in molecules involved in hepatocyte-specific, metabolic pathways (Fig. 2C) for xenobiotics by cytochrome P450 (Figure S3), drugs, retinol and all-trans-retinoic acid, amino acids, steroids.

Based on the literature^6–9^, we established a list of genes related to hepatocyte senescence (that blocks hepatocyte replication) and hepatic progenitor cell (HPC) richness (that can increase in case of blockade of hepatocyte replication) to examine whether these molecules were differentially expressed between NR and R. In addition, markers of hepatocyte proliferation and major hepatocyte fate determinants were included in the gene list. Livers from Indian NR over-expressed *CDKN1A* and *CDKN2A* (encoding p21 and p16, respectively) that are markers of hepatocyte senescence (Table S6). Indian NR patients demonstrated increased expression of some genes (*KRT19*, *SPP1*) from HPC. *MKI67* (marker of hepatocyte proliferation) and *HNF1A* and *HNF4B* (major hepatocyte fate determinants) were not differentially expressed between NR and R.

#### French livers

The French cohort had 207 DEGs between NR and R; 65 were over-expressed and 142 under-expressed (Table S5A and B). Over-expressed genes were enriched in GO terms “Hemoglobin complex”, “Oxygen transport”, “Heme binding” (Table S5C). Under-expressed genes were enriched in GO terms and KEGG pathways related to mitotic cell cycle, G1/S transition to mitotic cell cycle, DNA replication (Tables S5D and E). In the French livers genes related to senescence, HPC and hepatocyte proliferation were not differentially expressed between NR and R (Table S6).

#### Comparative anlysis

Livers of French non-responders exhibited enrichment in hemoglobin-related genes, in particular, hemoglobin-coding genes such as *HBB*, *HBA1*, *HBD*, *HBG2*, and *HBE1*. Examination of hemoglobin-erlated genes in the Indian non-responder’s livers also identified an over-expression of *HBM* and *HBQ1*. It is interesting to note that *HBM*, *HBQ1*, *HBD*, *HBE1* and *HBG2* are produced in fetal livers.

### NR3C1 Transcripts and Glucocorticoid receptor protein (GR) profile

#### NR3C1 transcript variation

**Figure 3:**
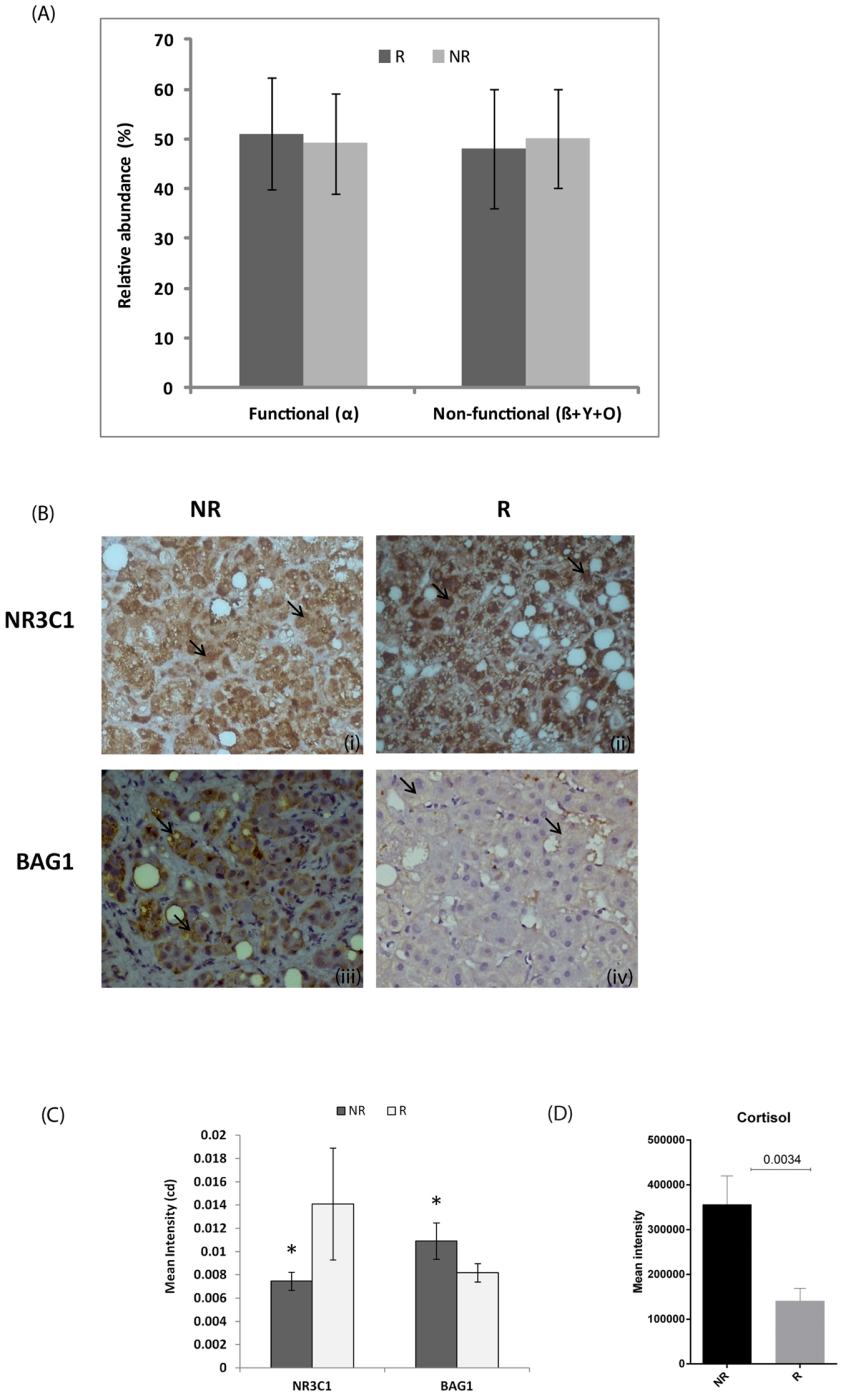
Expression of NR3C1 transcript variants and its protein among Indian NR and R. (**A**) The functional and non-functional transcript variants in R and NR were not significantly distinct. (**B**) The GR degrading protein BAG1 correlates with reduction in the NR3C1 in liver biopsy sections (40×): (i) and (ii) are biopsy sections from NR and R respectively, stained with NR3C1 antibody; (iii) and (iv) are sections from NR and R respectively stained with BAG1 antibody. Arrows indicate the presence of respective proteins in the cells. (**C**) Quantitation of the chromogenic signal from the anti-BAG1 and anti-NR3C1 antibodies confirms an increase in BAG1 and significant reduction in GR protein among NR. (**D**) Histogram showing the comparative values of cortisol (in terms of mean intensities as measured by mass spectrophotometer) for NR and R. *Indicates a significance value of P < 0.05.

Alternative splicing of the *NR3C1* transcript gives rise to transcripts coding for three receptor isoforms— GRα, GRβ, and GRγ^10^. In Indian livers, overall *NR3C1* expression did not differ between NR and R. In both NR and R expressed 13 *NR3C1* transcripts including 5 for GRα, 1 for GRβ, 1 for GRγ and the remaining 6, having non-coding status. However, the hepatic distribution of “functional” (GRα) versus “non-functional” (GRβ, GRγ, and non-coding transcripts) did not significantly differ between NR and R (Fig. 3A). In French patients, there was no difference in overall *NR3C1* expression between NR and R. Both NR and R expressed in the same proportion the 3 *NR3C1* transcripts, including 2 for GRα and 1 with unknown function (Figure S4).

#### Altered post-translational regulation of GR, NLRP3 and BAG1

Leukemia cells over-expressing *CASP1* (encoding caspase 1) and its activator, *NLRP3* (encoding NLR family, pyrin domain containing 3), do not respond to glucocorticoids because over-expressed caspase 1 cleaves the transactivation domain of the GR protein^11^. We explored this hypothesis in Indian and French patients. However, this mechanism of non-response to glucocorticoids was not observed as the hepatic *NLRP3* and *CASP1* were not differentially expressed between NR and R in the two populations. We found hepatic *BAG1* (encoding BCL2 associated athanogene 1) was over-expressed in NR relative to R in Indian (Table S4A). The protein BAG-1 has been demonstrated to take GR for protein degradation^12^. Thus, we asked if up-regulation of this gene in Indian NR was associated with decreased *NR3C1* gene product. Immunostaining of biopsies with anti-GR antibody demonstrated a reduced protein expression in hepatocytes from NR (Fig. 3B(i–iv) and C). Serial sections of the same biopsies showed an increase in the *BAG1* staining, suggesting to BAG1-regulated decrease in GR availability. Interestingly, *BAG1* was not differentially expressed in the French patients (Table S5F), suggesting that *BAG1*-mediated regulation of GR may not be universal.

#### Identification of proliferation related gene targets of glucocorticoid receptor

Finally, we asked if the glucocorticoid treatment may impact expression of genes involved in hepatocyte proliferation. Thus, we manually queried the Gene Ontology database (http://www.geneontology.org) for genes involved in cell proliferation, mitosis or cell cycle, and established a list of 1188 genes (Table S7A). Next we queried this gene set in Nuclear Receptor Signaling Atlas (NURSA) database (https://www.nursa.org) for glucocorticoid targets. We found 23 genes to be modulated by glucocorticoids (Table S7B), irrespective of the tissue. Amongst these, two genes *CDC20* and *CDK6* (both being pro-proliferative), are known to be modulated (down-regulated) by the glucocorticoid receptor in the liver.

## Discussion

Because of the lack of accurate animal models of SAH, translational research in livers from patients with SAH is of major interest. This study enrolled a series of patients with SAH in India and in France, using similar inclusion criteria. It is the first study to perform extensive gene profiling in patients with SAH, at baseline, i.e., before any glucocorticoid therapy. Our first objective was to identify DEGs between livers and PBMCs, both tissues being taken in the same patients, in each cohort. Two different techniques were used: RNA-Seq in Indian patients and expression microarray in French patients. Although, the number of DEGs differed between the Indian and French patients (1,663 vs. 8,872, respectively), this difference did not seem to be a result of different strategies adopted by two techniques to capture transcripts. Indeed, additional experiments in French livers showed an excellent correlation between microarray and RNA-seq, suggesting that differences in DEGs between Indian and French livers were related to “intrinsic” differences in gene expression between livers. Moreover, the functional analysis of DEGs between livers and PBMCs provided similar results in the two series of patients confirmed that the two techniques used were able to capture liver-specific genes. In addition, in Indian and French patients, we found a set of cytokines, chemokines and cytokine receptors that were expressed in livers but not PBMCs, suggesting that these molecules could have been produced by livers cells or by “non-PBMC” cells (e.g., neutrophils) that have migrated from blood to the liver. Future studies should investigate the origin of these molecules and their role in liver in inflammation in patients with SAH.

Baseline liver transcriptome has never been compared between NR and R patients with SAH. In this study, we identified hepatic DEGs between NR and R, in the Indian and French series. In Indian livers, 1,106 genes were over-expressed and 96 under-expressed in NR relative to R. Functional annotation of over-expressed genes did not find any particular pathway enrichment, suggesting that these genes were related to diverse functions. Under-expressed genes enriched pathways involved in metabolism of xenobiotics and drugs. An important finding from analysis of DEGs between Indian NR and R was that NR over-expressed *CDKN1A* and *CDKN2A*, two established markers of hepatocyte senescence, i.e., irreversible block to hepatocyte replication, a hallmark of advanced liver diseases^7–9^. This is consistent with more marked hepatocyte senescence in Indian NR and subsequent blockade of the hepatocytes’ ability to regenerate themselves. Hepatocyte death which occurs in SAH, could not be compensated in NR by hepatocyte self-renewal because of hepatocyte senescence. However, in the context of hepatocyte senescence or defective replication, ductular reactions are expected to develop, characterized by the involvement of HPCs that express several biliary markers^7^. HPCs could give rise to functional hepatocytes. In Indian livers, there was no clear-cut difference in HPC proliferation. Among HPC markers, some were over-expressed (*KRT19*, *SPP1* [encoding osteopontin]) in NR but several, such as *EPCAM*, *SOX9*, *HNF1B*, *LGR5*, *ASCL2* were not differentially expressed between NR and R (Table S6). Livers from NR over-expressed *TNFRSF12A* (encoding Fn14, a crucial receptor for mitogenic stimulation of HPCs) but *TNFSF12* (encoding TWEAK, the agonist for Fn14) was not differentially expressed between NR and R. Finally, it is important to note that even if NR patients with SAH may exhibit features of HPC proliferation, these cells are unable to differentiate into functional hepatocytes^13^. This failure to differentiate into functional hepatocytes could explain the poor richness of livers of Indian NR in transcripts involved in very important hepatocyte functions such as metabolism of xenobiotics and drugs.

In French livers, 65 genes were over-expressed and 142 under-expressed, in NR relative to R. Over-expressed genes were mostly related to hemoglobin; underexpressed genes were involved mitotic cell cycle, G1/S transition to mitotic cell cycle, DNA replication, suggesting defective proliferative capacity of hepatocytes. Interestingly, unlike Indian NR, French NR did not exhibit the features of hepatocyte senescence and HPC proliferation.

Together these findings show that the landscape of DEGs is different between Indian NR and French NR. The diversity of landscape of DEGs between the two populations could be due to differences in genetic and environmental factors, and age. In addition, the score for Mallory-Denk bodies was significantly higher in Indian NR than in R, but this difference was not found between French NR and R. Liver fibrosis was more marked in French NR than in R while it was similar in Indian NR and R. Therefore, differences in liver pathology may contribute to differences in the landscape of DEGs between Indian and French patients. However, these findings should be interpreted with caution because of the small number of patients, in particular French patients.

Nevertheless, our findings in Indian and French patients converge on a decreased baseline ability to regenerate the liver, as a major feature that characterizes NR, relative to R. Our results comparing liver transcriptome between NR and R confirm and extend previous findings that have been obtained by using a less comprehensive approach to compare livers from NR to livers from cirrhotic patients without SAH^14^. Accordingly, the ideal objective of treatment for NR patients with SAH seems to be the stimulation of liver regeneration, for example using cell transplantation.

An interesting new finding of our study was that livers of French NR over-expressed 5 genes encoding different hemoglobins. Livers of Indian NR also over-expressed 2 genes coding for hemoglobin. These 2 genes as well as 3 of the 5 genes in the French livers are known to encode fetal hemoglobin; this should lead to future investigations.

Baseline liver inflammation is a hallmark of SAH^15^ and the reason why glucocorticoid therapy is used in this disease. In this study genes over-expressed in livers from Indian and French NR, were not enriched in GO terms or KEGG pathways related to the inflammatory, defense, innate immune responses, suggesting that there was no difference in baseline liver inflammation between NR and R.

An important part of this study was to investigate expression of the glucocorticoid receptor in livers from Indian patients with SAH. This receptor has three protein isoforms – α, ß and γ. The α isoform is the active form of the receptor, whereas β isoform does not bind glucocorticoids. GRγ is characterized by the insertion of an additional amino acid in the DNA binding domain and this decreases the transcriptional activation by the glucocorticoid receptor^16^. At baseline, in livers of Indian and French NR, we did not find a significant increase in the non-functional alternative spliced transcripts of *NR3C1* that could contribute to the lack of response to glucocorticoids. We further explored an alternative pathway for loss of GR in hepatocytes. In neurological diseases, BAG-1 has been shown to reduce the function of glucorticoid receptor^12, 17^. In Indian NR but not in French NR *BAG1* gene was over-expressed. Immunohistochemically, BAG-1 was significantly upregulated in Indian NR and associated with significant reduction in glucocorticoid receptor. Therefore, BAG-1 overexpression could contribute to non-response to glucocorticoid therapy in Indian patients but not in French patients. In addition, in Indian patients, baseline plasma cortisol levels were higher in NR than in R. These high cortisol levels may be responsible for a saturation of GR by endogenous ligand, decreasing receptor availability for the exogenous ligand^18^.

Another potential mechanism of lack of response to glucocorticoid could be the defect in the capacity of liver regeneration observed in non-responders. This hypothesis should be further evaluated.

Our systematic analysis on glucocorticoid target genes associated with hepatocyte proliferation suggests that glucocorticoids could have a negative impact on liver regeneration. Of note, this study was not designed to investigate the effect of glucocorticoid on liver regeneration. Ideally, a comparison of liver transcriptome before and after 7 days of glucocorticoid therapy should be done. This however, would require two liver biopsies, which may not be ethically justified.

A limitation of our study is that it is a pilot study with small sample size. Accordingly, our results should be interpreted with caution and require a larger study cohorts for confirmation. Moreover, for ethical reasons, we did not have access to liver and PBMCs of healthy subjects or of heavy drinkers without significant liver disease, and therefore could not compare with our patient samples.

In conclusion, this study shows for the first time, that the baseline transcriptome tend to differ between NR and R, in Indian as well as French patients with SAH. Our results suggest defective capacity of the liver for regeneration in SAH patients, as a distinctive phenomenon that underlies non-response to glucorticoid therapy. Therefore, therapies stimulating liver regeneration may be of benefit to patients who do not respond to glucocorticoids. Finally, we found that the non-response to glucocorticoids could not be explained by a decrease in overall *NR3C1* transcript expression level or altered *NR3C1* splicing.

## Patients and Methods

### Indian patients

In a cross-sectional study, consecutive 102 patients with clinical and analytical features of severe alcoholic hepatitis were shortlisted at the Institute of Liver and Biliary Sciences, New Delhi between October 2013 and March, 2015. Patients having gastrointestinal hemorrhage in the last 15 days, moderate to severe ascites, hepatocellular carcinoma or other forms of malignancy, serological marker positivity for HBV, HCV, HIV or peptic ulcer. All the patients were screened for infection and sepsis before enrollment with blood cultures, urine cultures, procalcitonin levels, and chest X-ray. At enrollment, none of the patients had active infection or recent infection and everyone received antibiotics for prophylaxis. Transjugular liver biopsy was performed to confirm histological evidence of severe alcoholic hepatitis. Fifty-four patients were characterized as having cirrhosis and alcoholic hepatitis histologically and having Maddrey’s discriminant function (MDF) ≥32, recent onset of jaundice, history of long standing alcoholism continued till last 30 days of onset of jaundice and significantly raised AST/ALT. Baseline demographic profiles were recorded and blood samples were collected before the start of glucocorticoid therapy, prednisolone 40 mg/day. The laboratory staff processing the samples was blinded about the clinical details. All patients were managed according to the standard of care including intensive care monitoring, high calorie diet (35–40 cal/kg/day), intravenous albumin, broad-spectrum antibiotics, and glucocorticoid therapy for 7 days. Severity of liver disease was assessed by MDF, Child-Pugh (CP), and Model of End Stage Liver Disease (MELD) scores, at enrollment and at follow-up. The response to glucocorticoid therapy was assessed at day 7, by calculating the Lille score^2^. The Institutional Ethics Review Board (ILBS) approved the study vide protocol number NCT01820208 and an informed consent was taken from all patients enrolled for the study prior to sample collection. For the French patients, he protocol was approved by the French Ethics Committee (Comité de Protection des Personnes III, N° 2014-Aà1354–43). The patient characteristics for the French patients is provided in Supplemental methods. All the experiments were performed in accordance with the relevant guidelines and regulations.

Of the 54 patients with liver biopsy who underwent glucocorticoid therapy, 38 were R and 16 NR. While all 16 NR were included, matched 16 responders from the total 38 responders were also included in the study. There was no difference between the 16 responders included in the analysis and the remaining 22 not-included as far as their baseline characteristics were concerned. The baseline clinical characteristics of the 16 NR and 16 R are shown in Table S1.

After the quality control check, the samples were processed for library preparation according to the workflow defined in the Supplemental methods.

### Transcriptome analysis

We used highthroughput RNA-Seq based on Illumina HiSeq platform (for Indian patients) and GeneChip® Human Transcriptome Array (HTA) 2.0 (Affymetrix) (for French patients) to analyze the transcriptomes. Full description of the techniques used is mentioned in Supplemental Methods. Hereafter, *NR3C1* is used to refer to the glucocorticoid receptor gene and GR to represent its protein. Further details about the next generation sequencing in the Indian and the French patient livers and the microarray in the French patient livers is provided in the Supplementary Methods.

### Differential gene expression analysis

For differential gene expression analysis in the two cohorts, normalized signal values were converted to log-fold change before performing the analysis. Post-analysis and identification of genes that showed 1.5-fold change in expression; the value were reverted back to fold change for better understanding and interpretation. Over- and under-expressed gene sets were identified after considering only those genes that showed a significant variation of p < 0.05. Differentially expressed genes (DEGs; i.e., between livers and PBMCs or between NR and R) were analyzed with Database for Annotation, Visualization, and Integrated Discovery (DAVID) 6.8 Beta 2003–2016 (david.abcc.ncifcrf.govhome.jsp)^19, 20^, to query the open source databases such as Gene Ontology (www.geneontology.org), Kyoto Encyclopedia of Genes and Genomes (KEGG, www.genome.ad.jp/kegg/)^14^. In addition, for analysis of DEGs between livers and PBMCs DAVID was used to query UP_TISSUE.

### Immunohistochemical characterization

The biopsy specimens obtained from the Indian patients were stained with hematoxylin and eosin stains, Masson trichrome stain for histological assessment as per previous published protocol^21^. Trans-jugular liver biopsy tissues were cut to 3–4 μm thickness and were taken on poly-l-lysine coated slides. For immunohistochemical analysis of GR and BAG-1 in the biopsy samples, previously published protocol of Maras *et al*.^22^ was followed. GR polyclonal antibody (PB9232) was used at 1:200 titre (Boster Biologics, Pleasanton, CA, USA). This antibody is raised in rabbit against human GR recombinant protein (position: M1-D373) cloned in *E*. *coli*. The BAG-1 antibody (PA1392) was used at 1:50 titre (Boster Biologics, Pleasanton, CA, USA). This antibody is raised in rabbit against the human BAG-1 epitope DTVEQNICQETERLQSTNFALAE (322–344 aa). The intensity of the staining/labeling was measured using NIS-Elements AR ver4.0 (Nikon, USA). Background noise was removed and the intensities were normalized before comparison between groups.

### Cortisol estimation

Protocols defined by Boudah *et al*.^23^ were followed for measuring cortisol in plasma samples of patients. Briefly, after UHPLC/MS analysis raw data was processed by automatic peak detection software and were annotated using Camara software^24^. Identified features were than annotated based on the ESI-MS database (SPI-MS/MS database for metabolites- CEA, Saclay, France) and publically available metabolites database [KEGG, www.genome.jp/kegg/; HMDB^25^, METLIN^26^]. The relative abundance of the associated feature at 384.088 m/z and retention time of 7.26 min (cortisol) was measured and compared between the groups.

### Statistical analysis

Due to the pilot nature of this study and thus we did not estimate the sample size in different study groups based on power analysis. For RNA-seq in the Indian and the French study groups, ANOVA and Student t-test were used respectively, due to different number of biological replicates in each group. For microarray analysis, differential gene expression was assessed using an unpaired Student t test to compare gene intensities in the different biological replicates. Genes were considered significantly regulated when fold-change was be ≥1.5 and *P* ≤ 0.05 for both RNAseq and microarray.

## Electronic supplementary material

Supplementary Material

Supplementary Table S1

Supplementary Table S2

Supplementary Table S3

Supplementary Table S4

Supplementary Table S5

Supplementary Table S6

Supplementary Table S7
