# Supplementary material for: Pre-therapy liver transcriptome landscape in Indian and French patients with severe alcoholic hepatitis and steroid responsiveness
Source: Sci Rep. 2017 Jul 28;7:6816. doi: 10.1038/s41598-017-07161-4 (PMC5533759; doi:10.1038/s41598-017-07161-4)
Supplement: Supplementary file 1 — Supplementary Material [file 41598_2017_7161_MOESM1_ESM.pdf]

SREP 16-52023 R1

Supplementary Material

Pre-therapy liver transcriptome landscape in Indian and French patients with severe alcoholic hepatitis and steroid responsiveness

Shvetank Sharma, Jaswinder S. Maras, Sukanta Das, Shabir Hussain, Ashwani K. Mishra, Saggere M. Shasthry, Chhagan B. Sharma,Emmanuel Weiss, Laure Elkrief, Pierre-Emmanuel Rautou, Hélène Gilgenkrantz, Sophie Lotersztajn,Valérie Paradis, Pierre de la Grange, Christophe Junot, Richard Moreau, Shiv K. Sarin

Table of Content

Supplementary Patients and Methods

Supplementary Tables

Table S1: Baseline characteristics, including liver pathology, of 32 Indian and 8 French patients with severe alcoholic hepatitis. Characteristics are given according to the response to corticosteroids.

Table S2: Paired comparison of baseline transcriptome between livers and corresponding PBMCs, in Indian patients with severe alcoholic hepatitis: list of differentially expressed genes.

Table S3: Paired comparison of baseline transcriptome between livers and corresponding PBMCs, in French patients with severe alcoholic hepatitis. (A) List of differentially expressed genes between livers and corresponding PBMCs. (B) Hierarchical clustering of the top 500 differentially regulated genes in livers vs. correspondings PBMCs. (C) Functional classification of genes that were overexpressed in livers as compared to corresponding PBMCs. (D) Functional

classification of genes that were under-expressed in livers as compared to corresponding PBMCs.

Table S4: Baseline liver transcriptome landscape in Indian non-responders and responders to glucocorticoids. (A) List of hepatic genes that were differentially regulated at baseline. (B) List of hepatic genes that were not significantly differentially regulated.

Table S5: Baseline liver transcriptome landscape in Indian non-responders and responders to glucocorticoids. (A) List of genes that were differentially regulated between non-responders and responders. (B) Hierarchical clustering of regulated genes. (C) Enrichment analysis of overexpressed genes. (D) Enrichment analysis of under-expressed genes. (E) Example of the KEGG pathway "Cell cycle" which was enriched in genes that were under-expressed in livers from non-responders. (F) List of genes that were not differentially expressed between non-responders and responders.

Table S6: Expression of genes related to hepatocyte senescence and hepatic progenitor cell (HPC) richness, in livers from Indian and French patients with severe alcoholic hepatitis (SAH), according to the response to glucocorticoids (NR vs R).

Table S7: Identification of genes involved in proliferation and their potential targeting by glucocorticoids. (A) List of 1,188 genes involved in cell proliferation, mitosis or cell cycle, according to Gene Ontology database (http://www.geneontology.org). (B) Identification of 23 genes whose expression has been shown to be modulated by glucocorticoids. These genes were identified by quering Nuclear Receptor Signaling Atlas (NURSA) database (https://www.nursa.org) with the list of 1,188 genes shown in Table 7A.

Supplementary Figures

Figure S1: Representative coverage of reads in selected up and downregulated genes (A) *SLC27A4* and (B) *CYP3A5* respectively. The blocks in the Refseq (blue depicts exons (E1, E2 and so forth). The y-axis represents the number of reads recorded for a given site.

Figure S2: Scatter plot showing the individual correlation between the expression microarray and RNA-Seq gene expression profile in the liver of 4 French patients. Note the strong correlation between the two methods in capturing gene expression signals.

Figure S3: KEGG pathways for metabolism of xenobiotics by cytochrome P450 (hsa00980) associated with unde-expressed geneset in NR. The genes that were downregulated in the given pathway are highlighted in red[1,2,3].

Figure S4: *NR3C1* transcript overall expression and splicing in livers from responders and non-responders in the French series of patients with severe alcoholic hepatitis. (A) EASANA visualization: Representation of the gene structure and probe intensity level for the NR3C1 gene. (B) List of potential expressed transcripts: The second column describes alternative events described by transcript and the third column indicates the predicted proportion of transcripts based on probe expression. (C) Predicted protein products: Graphical representation of the transcript exon structure with UTR region in blue and coding sequence in pink. Protein domain legend: Violet => “Zinc finger, nuclear hormone receptor-type”; green => “Glucocorticoid receptor”; light green => “Streroid hormone receptor”; blue => “Nuclear hormone receptor, ligand-binding, core”.

Supplementary Patients and Methods

Patients

French patients

The protocol was approved by the French Ethics Committee (Comité de Protection des Personnes III, N° 2014-Aà1354-43). Eight patients with histologically proven cirrhosis with severe alcoholic hepatitis were enrolled after they gave informed signed consent to participate. These patients did not have received prior corticosteroid therapy. After baseline samples were obtained (including liver-biopsy specimens via the tranjugular route), corticosteroid therapy was given according to a protocol similar to that used for Indian patients. The response to corticosteroids was assessed at day 7 according to Lille classification. Treament was pursued until day 28 in responders and stopped in non-responders. There were no significant differences at baseline between responders and non-responders in terms of severity of liver disease and other characterisitics (Table S4).

Methods

Next Generation Sequencing (Indian cohort)

The study groups were randomized before sending for analysis. Total RNA was extracted from liver biopsies of patients using the Trizol method (Invitrogen, Carlsbad, California, USA). Equal amounts of DNA-free RNA were used to generate cDNA library using iScript cDNA Synthesis Kit #1708891 (BioRad, Hercules, California, USA), according to the manufacturer's instructions. Samples were processed by NextGenBio Life Sciences (New Delhi, India), for downstream sample preparation and analysis, which was performed following the previously published strategy[4]. The samples were processed as follows. After isolation of total RNA , their

RIN values were measured on a Agilent 2100 Bioanalyzer system. All the samples were found to be aving RIN value of higher than 7.This RNA was used to prepare Illumina mRNA library using TruSeq mRNA library preparation kit version 3.0 (Illumina, USA). The prepared library was then analyzed on Agilent 200 Bioanalyzer system for assessing the quality of the library. After all the libraries were analyzed on Bioanalyzer and all of them passed QC. 10 samples were indexed using unique barcodes and multiplexed together in a single pool. Illumina clusters were generated and were loaded onto Illumina Flow Cell on Illumina HiSeq 2000 instrument and sequencing was carried out using 2x100bp paired end chemistry.

Sequencing revealed an average mapping of 72.5% and 75% for R and NR respectively. The minimum reads were 23x106 and maximum were 74x106, with an average 36.9x106 reads/sample. After Sequencing, raw data was demultiplexed for each individual samples & their QC performed using FastQC (http://www.bioinformatics.babraham.ac.uk/ projects/fastqc/) and checked for reads containing less than Q20 phred quality score. The reads containing less than Q20 phred quality scored were filtered using FASTX TOOLKIT (http://hannonlab.cshl.edu/fastx_toolkit/). After quality filtering, the data generated per sample was in the range of 2.7 GB to 4.0 Gb per sample. The raw data of all the samples was mapped on Human Reference Sequence GRCh37.p13 build using Bowtie2[4]. Experimental grouping related to experimental conditions were defined after which quantification of expression levels of genes, exons & transcripts was done by counting the number of reads mapped to each gene or transcript. The differential expression was calculated between the sample groups using normalized read count values which are log transformed raw read count values was done using DESeq[5] package. And Inter & Intra group differential expression was calculated

using custom in-house script on normalized expression values of every gene including Fold Change & Log Fold Change values.

Expression Microarray (French patients)

Liver biopsy specimens taken at enrollment as well as PBMCs taken at enrollment and on day 7 of corticosteroid therapy have been used for expression microarray according to the protocol.

RNA isolation

Total RNA was extracted with TRIzol® Reagent (Invitrogen) then quantified using a ND-1000 NanoDrop spectrophotometer (NanoDrop Technologies) and purity/integrity was assessed using disposable RNA chips (Agilent RNA 6000 Nano LabChip kit) and an Agilent 2100 Bioanalyzer (Agilent Technologies, Waldbrunn, Germany). Only RNA preparations with $8.9 \leq$ RIN ≤ 9.8 were further processed for analysis (microarrays, RT-qPCR).

Expression Microarray

As previously described[6], GeneChip Human Gene 2.0 ST arrays (Affymetrix, Santa Clara, CA) were hybridized by GenoSplice technology (www.genosplice.com) according to the Ambion WT protocol (Life technologies, France) and Affymetrix (Santa Clara, CA) labelling and hybridization recommendations. Briefly, 100 ng of total RNA were first mixed with bacterial transcripts and the mixture was reverse transcribed into cDNA. After synthesis of double-stranded cDNA, an in vitro transcription reaction was conducted overnight. Resulting amplified cRNA was reverse transcribed into sense DNA incorporating dUTP. This single stranded DNA was treated with a combination of uracil DNA glycosylase and apurinic/apyrimidinic endonuclease 1. DNA fragments was biotin-labelled by terminal deoxynucleotidyl

transferase. Targets was prepared according Affymetrix recommendations for hybridization of exon arrays. Microarrays was hybridized, washed and scanned using Affymetrix instruments. Raw data was controlled with the Expression console (Affymetrix). Briefly, all "pos_vs_neg_auc" value was above 0.8 (detection of positive controls against the false detection of negative controls). Hybridization Quality was checked using bacterial spikes expression and Labeling Quality was checked using polyA-control RNAs expression. Only genes expressed in at least one compared condition was analyzed.

Hierarchical Clustering: The distance from the gene signal in a given sample to the corresponding average in all the samples was calculated for each regulated gene. Corresponding values was displayed and clusterized with MeV4.6.2 from The Institute of Genome Research using Pearson correlation and average linkage clustering.

Functional analysis: KEGG and REACTOME pathways were queried using Database for Annotation, Visualization, and Integrated Discovery (DAVID) Bioinformatics Resources 6.7, an online graph theory evidence-based method to agglomerate heterogeneous and widely distributed public databases (http://david.abcc.ncifcrf.gov/home.jsp).

Next Generation Sequencing (French patients)

Sequencing, data quality, reads repartition (e.g., for potential ribosomal contamination), and insert size estimation were performed using FastQC, Picard-Tools, Samtools and rseqc. Reads were mapped using STARv2.4.0 [PMID: 23104886] on the hg19 Human genome assembly. Gene expression regulation study was performed as already described [PMID: 26584541; 27174676; 28009274].

Briefly, for each gene present in the Human FAST DB v2016_1 annotations, reads aligning on constitutive regions (that are not prone to alternative splicing) were counted using featureCounts v1.5.0-p2. Based on these read counts, normalization and differential gene expression were performed using DESeq2 [PMID: 25516281] on R (v.3.2.5). Only genes expressed in at least one of the two compared experimental conditions were further analyzed. Genes were considered as expressed if their rpkm value was greater than 97.5% of the background rpkm value based on intergenic regions. Results were considered statistically significant for p-values ≤ 0.05 and fold-changes ≥ 1.5.

Supplementary Results

High cortisol levels in Indian NR

Cortisone produced in the adrenal is transported to hepatocytes, where it is converted to active cortisol by 11-beta-hydroxysteroid dehydrogenase 1 (*HSD11B1*) in the hepatocytes[7]. Interestingly, the expression levels of *HSD11B1* did not differ between NR and R (Table S3) suggesting no alteration in hepatic cortisone conversion. Level of total cortisol in plasma of the patients revealed significant difference between the NR and R (3.5×10^5 versus 1.5×10^5 arbitrary units respectively, P=0.0034) (Figure 3D), indicating to possible saturation of non-degraded GR and hence non-response to glucocorticoid therapy.

References:

1. Kanehisa, M., Furumichi, M., Tanabe, M., Sato, Y., and Morishima, K.; KEGG: new perspectives on genomes, pathways, diseases and drugs. Nucleic Acids Res. 45, D353-D361 (2017).
2. Kanehisa, M., Sato, Y., Kawashima, M., Furumichi, M., and Tanabe, M.; KEGG as a reference resource for gene and protein annotation. Nucleic Acids Res. 44, D457-D462 (2016).
3. Kanehisa, M. and Goto, S.; KEGG: Kyoto Encyclopedia of Genes and Genomes. Nucleic Acids Res. 28, 27-30 (2000).
4. Langmead B, Salzberg S. Fast gapped-read alignment with Bowtie 2. (2012) Nature Methods. 2012, 9:357-359.
5. Anders S. and Huber, W. (2010). Differential expression analysis for sequence count data. Genome Biology **11**.
6. Gandoura S, Weiss E, Rautou PE, Fasseu M, Gustot T, Lemoine F, Hurtado-Nedelec M, Hego C, Vadrot N, Elkrief L, Lettéron P, Tellier Z, Pocidalo AM, Valla D, Lebrec D, Groyer A, Monteiro RC, de la Grange P, Moreau R. Gene- and exon-expression profiling reveals an extensive LPS-induced response in immune cells in patients with cirrhosis. J Hepatol 2013;58:936-48.
7. Chapple, R. H. et al. Characterization of the rat developmental liver transcriptome. Physiol Genomics 45, 301-311, doi:10.1152/physiolgenomics.00128.2012 (2013).

Figure S1

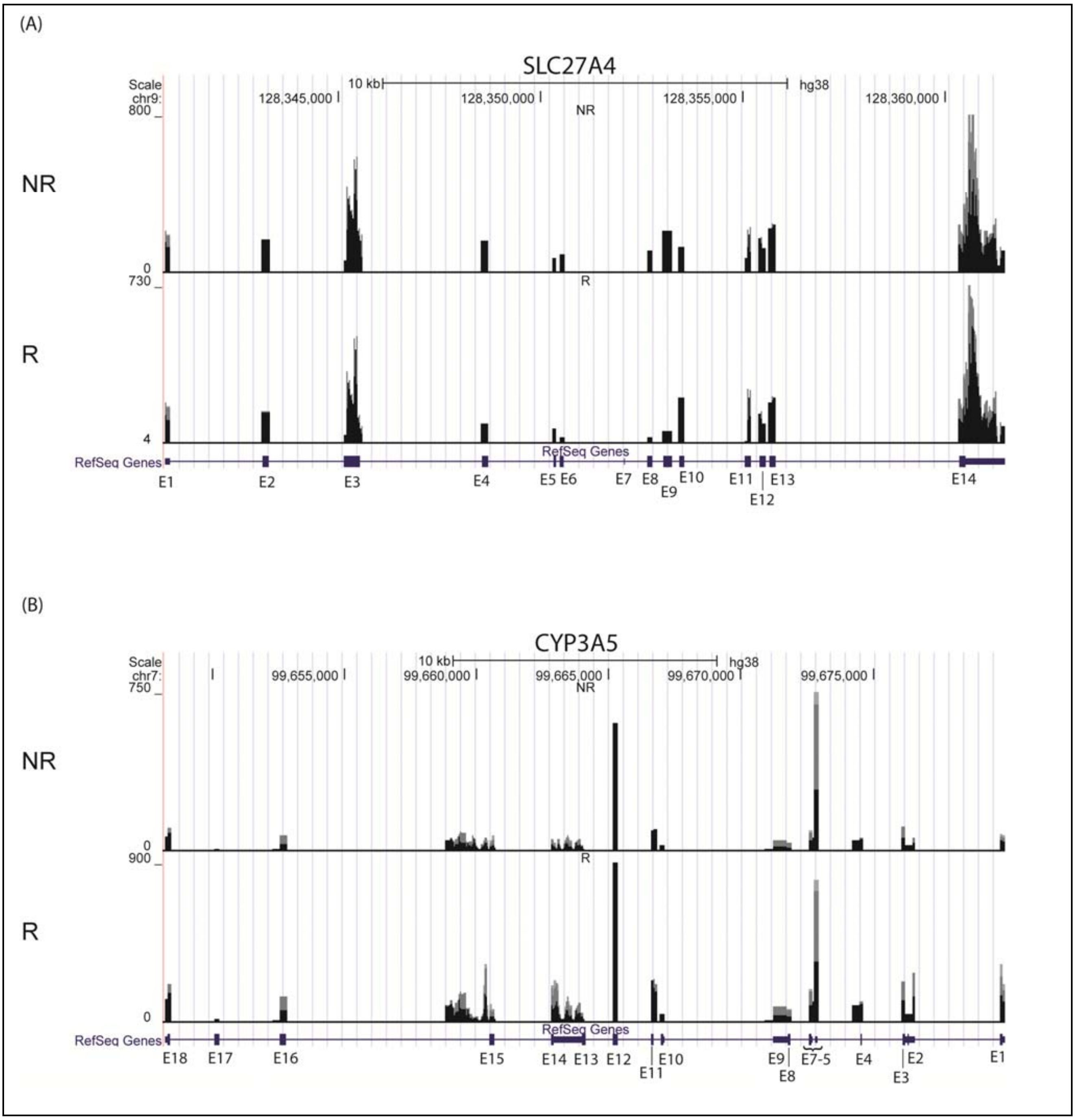
(A)
SLC27A4
Scale
chr9:
10 kb
hg38
128,345,000
128,350,000
128,355,000
128,360,000
NR
R
RefSeq Genes
E1 E2 E3 E4 E5 E6 E7 E8 E9 E10 E11 E12 E13 E14
(B)
CYP3A5
Scale
chr7:
10 kb
hg38
99,655,000
99,660,000
99,665,000
99,670,000
99,675,000
NR
R
RefSeq Genes
E18 E17 E16 E15 E14 E13 E12 E11 E10 E9 E8 E7-5 E4 E3 E2 E1

Figure S2

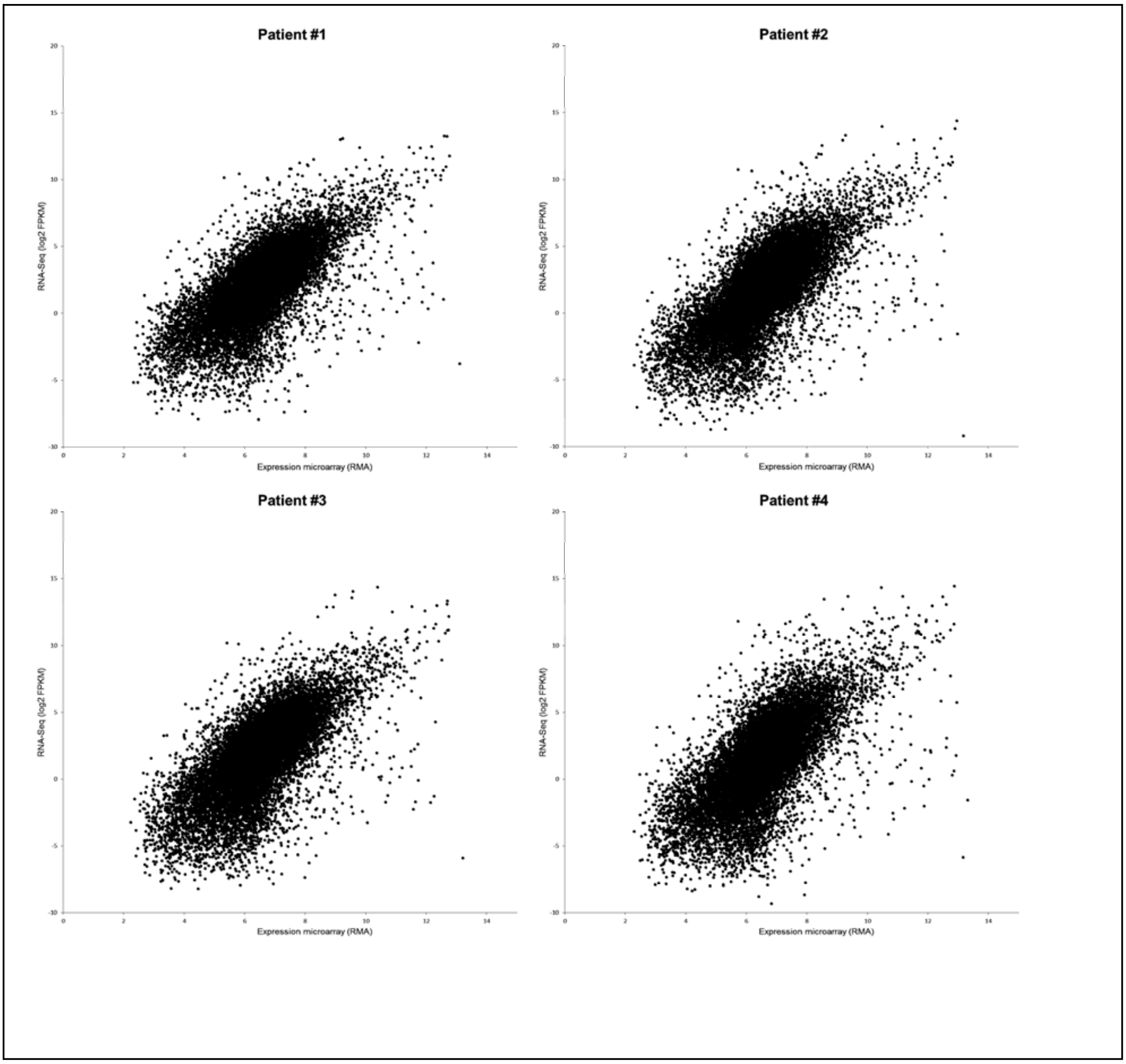
Patient #1
Patient #2
Patient #3
Patient #4
RNA-Seq (log2 FPKM)
Expression microarray (RMA)

Figure S3

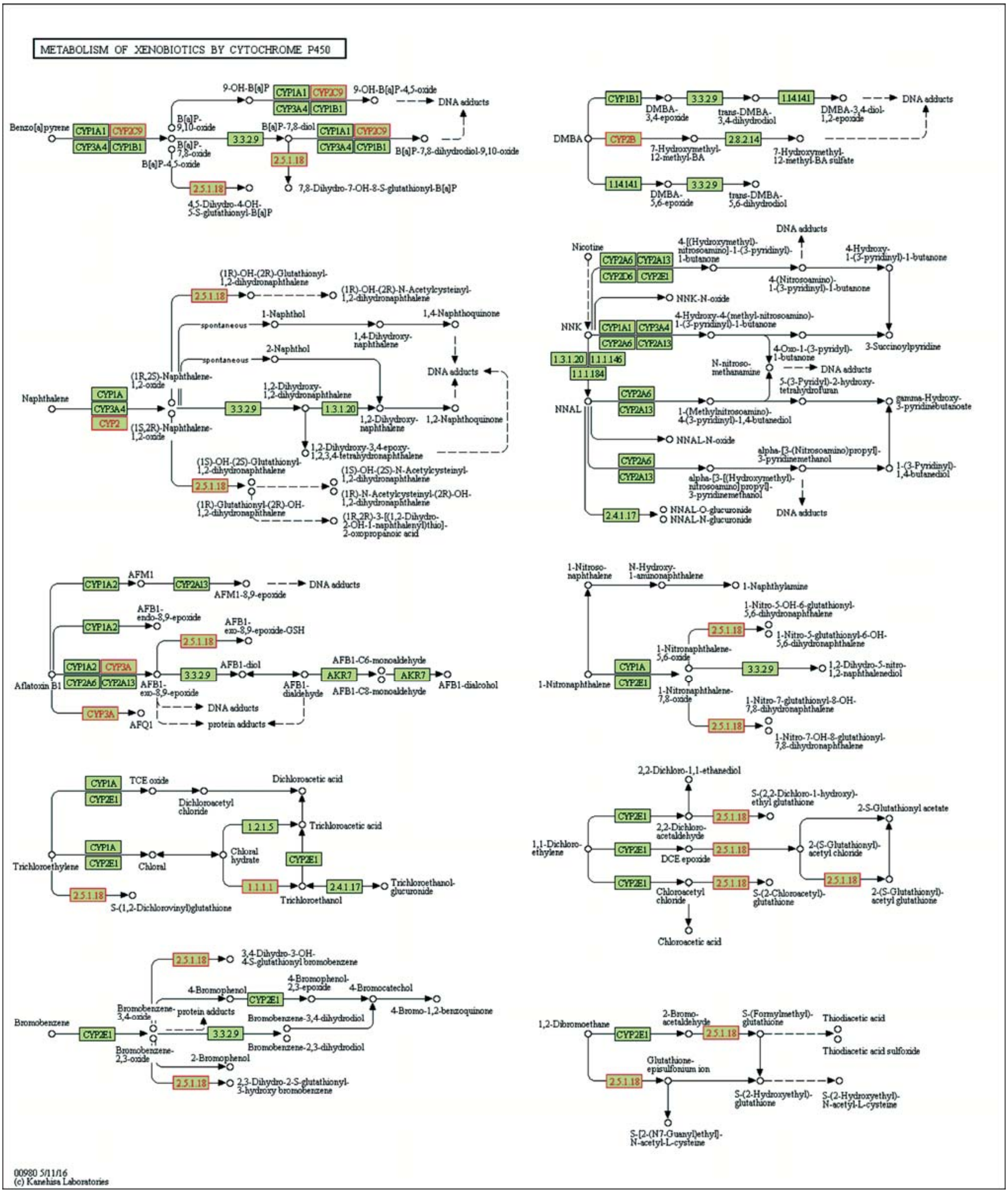
METABOLISM OF XENOBIOTICS BY CYTOCHROME P450
Benzo[a]pyrene
CYP1A1
CYP2C9
CYP3A4
CYP1B1
9-OH-B[a]P
9-OH-B[a]P-4,5-oxide
DNA adducts
B[a]P-9,10-oxide
B[a]P-7,8-oxide
B[a]P-4,5-oxide
B[a]P-7,8-diol
B[a]P-7,8-dihydrodiol-9,10-oxide
3.3.2.9
2.5.1.18
7,8-Dihydro-7-OH-8-S-glutathionyl-B[a]P
4,5-Dihydro-4-OH-5-S-glutathionyl-B[a]P
DMBA
DMBA-3,4-epoxide
trans-DMBA-3,4-dihydrodiol
DMBA-3,4-diol-1,2-epoxide
1.1.1.41
CYP2B
7-Hydroxymethyl-12-methyl-BA
2.8.2.14
7-Hydroxymethyl-12-methyl-BA sulfate
DMBA-5,6-epoxide
trans-DMBA-5,6-dihydrodiol
Naphthalene
CYP1A
CYP2
(1R,2S)-Naphthalene-1,2-oxide
(1S,2R)-Naphthalene-1,2-oxide
(1R)-OH-(2R)-Glutathionyl-1,2-dihydronaphthalene
(1R)-OH-(2R)-N-Acetylcysteinyl-1,2-dihydronaphthalene
spontaneous
1-Naphthol
2-Naphthol
1,4-Naphthoquinone
1,4-Dihydroxy-naphthalene
1,2-Dihydroxy-1,2-dihydronaphthalene
1.3.1.20
1,2-Dihydroxy-naphthalene
1,2-Naphthoquinone
1,2-Dihydroxy-3,4-epoxy-1,2,3,4-tetrahydronaphthalene
(1S)-OH-(2S)-Glutathionyl-1,2-dihydronaphthalene
(1S)-OH-(2S)-N-Acetylcysteinyl-1,2-dihydronaphthalene
(1R)-Glutathionyl-(2R)-OH-1,2-dihydronaphthalene
(1R)-N-Acetylcysteinyl-(2R)-OH-1,2-dihydronaphthalene
(1R,2R)-3-[(1,2-Dihydro-2-OH-1-naphthalenyl)thio]-2-oxopropanoic acid
Nicotine
CYP2A6
CYP2A13
CYP2D6
CYP2E1
4-[(Hydroxymethyl)-nitrosoamino]-1-(3-pyridinyl)-1-butanone
4-(Nitrosoamino)-1-(3-pyridinyl)-1-butanone
4-Hydroxy-1-(3-pyridinyl)-1-butanone
NNK
NNK-N-oxide
4-Hydroxy-4-(methyl-nitrosoamino)-1-(3-pyridinyl)-1-butanone
4-Oxo-1-(3-pyridyl)-1-butanone
3-Succinoylpyridine
N-nitroso-methanamine
5-(3-Pyridyl)-2-hydroxy-tetrahydrofuran
1.3.1.20
1.1.1.146
1.1.1.184
NNAL
1-(Methylnitrosoamino)-4-(3-pyridinyl)-1,4-butanediol
gamma-Hydroxy-3-pyridinebutanoate
NNAL-N-oxide
alpha-[3-(Nitrosoamino)propyl]-3-pyridinemethanol
alpha-[3-[(Hydroxymethyl)-nitrosoamino]propyl]-3-pyridinemethanol
1-(3-Pyridinyl)-1,4-butanediol
2.4.1.17
NNAL-O-glucuronide
NNAL-N-glucuronide
Aflatoxin B1
CYP1A2
CYP3A
AFM1
AFM1-8,9-epoxide
AFB1-endo-8,9-epoxide
AFB1-exo-8,9-epoxide-GSH
AFB1-exo-8,9-epoxide
AFB1-diol
AFB1-dialdehyde
AFB1-C6-monoaldehyde
AFB1-C8-monoaldehyde
AKR7
AFB1-dialcohol
AFQ1
protein adducts
1-Nitronaphthalene
1-Nitroso-naphthalene
N-Hydroxy-1-aminonaphthalene
1-Naphthylamine
1-Nitronaphthalene-5,6-oxide
1-Nitronaphthalene-7,8-oxide
1-Nitro-5-OH-6-glutathionyl-5,6-dihydronaphthalene
1-Nitro-5-glutathionyl-6-OH-5,6-dihydronaphthalene
1,2-Dihydro-5-nitro-1,2-naphthalenediol
1-Nitro-7-glutathionyl-8-OH-7,8-dihydronaphthalene
1-Nitro-7-OH-8-glutathionyl-7,8-dihydronaphthalene
Trichloroethylene
TCE oxide
Dichloroacetyl chloride
Dichloroacetic acid
1.2.1.5
Trichloroacetic acid
Chloral
Chloral hydrate
1.1.1.1
Trichloroethanol
2.4.1.17
Trichloroethanol-glucuronide
S-(1,2-Dichlorovinyl)glutathione
1,1-Dichloro-ethylene
2,2-Dichloro-1,1-ethanediol
2,2-Dichloro-acetaldehyde
S-(2,2-Dichloro-1-hydroxy)-ethyl glutathione
2-S-Glutathionyl acetate
DCE epoxide
2-(S-Glutathionyl)-acetyl chloride
Chloroacetyl chloride
S-(2-Chloroacetyl)-glutathione
2-(S-Glutathionyl)-acetyl glutathione
Chloroacetic acid
Bromobenzene
Bromobenzene-3,4-oxide
Bromobenzene-2,3-oxide
3,4-Dihydro-3-OH-4-S-glutathionyl bromobenzene
4-Bromophenol
4-Bromophenol-2,3-epoxide
4-Bromocatechol
4-Bromo-1,2-benzoquinone
Bromobenzene-3,4-dihydrodiol
Bromobenzene-2,3-dihydrodiol
2-Bromophenol
2,3-Dihydro-2-S-glutathionyl-3-hydroxy bromobenzene
1,2-Dibromoethane
2-Bromo-acetaldehyde
S-(Formylmethyl)-glutathione
Thiodiacetic acid
Thiodiacetic acid sulfoxide
Glutathione-episulfonium ion
S-(2-Hydroxyethyl)-glutathione
S-(2-Hydroxyethyl)-N-acetyl-L-cysteine
S-[2-(N7-Guanyl)ethyl]-N-acetyl-L-cysteine
00980 5/11/16
(c) Kanehisa Laboratories

Figure S4

(A)

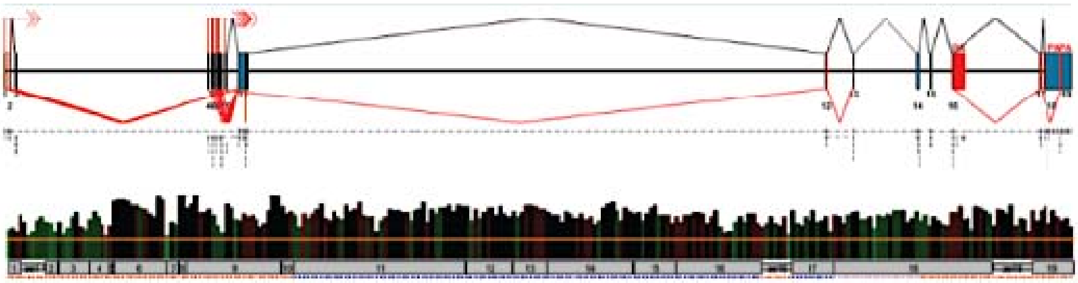

(B)

Transcript ID	Specific events	Predicted proportion
NM_001018076	*Start exon 6, retention intron 18*	≈75% (based on exon 6 expression)
NM_000176	*Start exon 9, retention intron 18*	≈15% (based on intron 18 expression)
NM_001204265	*Start exon 9, end within intron 16*	≈10% (based on intron 16 start expression)

(C)

Transcript ID	Exon content with protein domains	Comment (domains)
NM_001018076	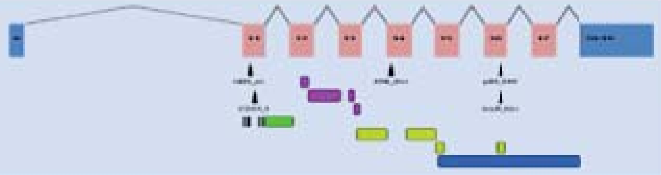	*ALL DOMAINS*
NM_000176	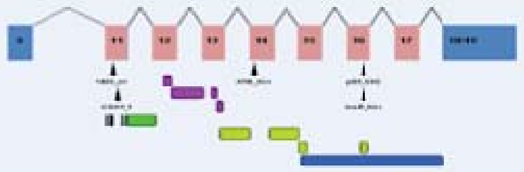	*ALL DOMAINS*
NM_001204265	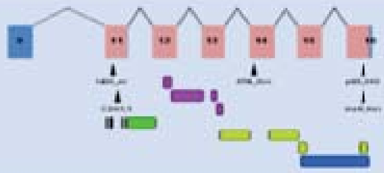	*NUCLEAR RECEPTOR AFFECTED*
